# Minimally invasive surgery in the correction of recurrent hallux valgus: a case series with 2‑year follow‑up

**DOI:** 10.1186/s12891-026-09946-z

**Published:** 2026-05-11

**Authors:** Gabriel Ferraz Ferreira, Isadora Michely Magalhães, Monique Ferreira Watari, José Venâncio Moreira Rebelato, Gustavo Araujo Nunes, Thomas Lorchan Lewis, Peter Lam, Robbie Ray, Gonzalo F. Bastías, Miguel Viana Pereira Filho

**Affiliations:** 1 Foot and Ankle Surgery Group, Orthopaedics and Traumatology Unit, Prevent Senior, São Paulo, Brazil; 2 State University of Campinas (UNICAMP), Campinas, São Paulo, Brazil; 3 Orthopaedics and Traumatology Unit, Prevent Senior, São Paulo, Brazil; 4 Foot and Ankle Unit, COTE Brasília Clinic, Brasília, DF Brazil; 5 Orthopaedic and Arthritis Specialist Centre, Chatswood, Sydney, Australia; 6 King’s Foot and Ankle Unit, King’s College Hospital London NHS foundation trust, London, England, UK; 7 Clínica Las Condes, Santiago, Chile; 8 Hospital del Trabajador, Santiago, Chile; 9 Head of Foot and Ankle Surgery Group, Orthopaedics and Traumatology Unit, Prevent Senior, São Paulo, Brazil

**Keywords:** Hallux Valgus, Osteotomy, Minimally Invasive Surgical Procedures, Reoperation, Retrospective Studies, PECA, MICA, META, Revision Bunion Surgery, Treatment Outcome, MOXFQ

## Abstract

**Objective:**

Recurrent deformity following surgical correction remains a complex and technically demanding condition. This study aimed to evaluate the clinical and radiographic outcomes of minimally invasive surgery (MIS) in patients undergoing revision procedures for recurrent hallux valgus (HV).

**Methods:**

This retrospective case series included 33 feet that underwent minimally invasive revision surgery using either the third generation Minimally Invasive Chevron-Akin (MICA) or the fourth-generation Metaphyseal Extra-Articular Transverse and Akin osteotomies (META), following failure of a previous hallux valgus correction performed by open or minimally invasive techniques. Clinical outcomes were assessed using the Manchester-Oxford Foot Questionnaire (MOXFQ), the Visual Analogue Scale (VAS) for pain, and patient satisfaction. Radiographic parameters and complications were recorded and statistically analysed using R software.

**Results:**

Both techniques proved effective in angular correction, with significant reductions in the hallux valgus angle (HVA) and intermetatarsal angle (IMA) (*p* < 0.001), as well as in bone and soft-tissue forefoot width (*p* < 0.05). Significant improvements were also observed in all three MOXFQ domains (*p* < 0.001) and in VAS scores (*p* < 0.001). Subgroup analysis revealed no statistically significant differences between MICA and META in either radiographic or clinical outcomes related to pain and function (*p* > 0.05).

**Conclusion:**

Third- and fourth-generation minimally invasive surgical techniques have proven effective in correcting radiographic parameters and improving pain and functional scores in patients undergoing revision for recurrent hallux valgus, with a low rate of complications.

**Level of evidence:**

Level IV, retrospective case series.

**Supplementary Information:**

The online version contains supplementary material available at 10.1186/s12891-026-09946-z.

## Introduction

Minimally invasive surgery (MIS) techniques are increasingly being utilised for the correction of hallux valgus, including in cases of recurrence, although most published data focus on primary surgery with revision cases excluded [1–8].

Recently, several robust studies have demonstrated the ability of third generation minimally invasive Chevron-Akin (MICA) and fourth-generation Metaphyseal Extra-Articular Transverse and Akin osteotomies (META) to correct hallux valgus with a low complication rate, while providing superior outcomes compared with open surgery in terms of perioperative pain and scar length [2, 4–6].

In addition, both techniques have demonstrated the ability to correct first metatarsal pronation, proving effective in reducing recurrence, which has been associated with failure to reposition the sesamoids and inadequate correction of rotational deformities [9, 10].

Thus, minimally invasive techniques for hallux valgus correction can now be considered an appropriate and effective treatment option for primary hallux valgus correction, no longer regarded as experimental procedures [2, 11, 12].

However, there is a lack of support in the literature regarding studies that have employed both techniques in the treatment of recurrent hallux valgus. Lewis and colleagues reported a series of 34 patients with recurrent hallux valgus who underwent corrective surgery using the META technique, demonstrating satisfactory clinical and radiographic outcomes [13].

The objective of this study was to evaluate a series of patients with recurrent hallux valgus who underwent minimally invasive correction using third- or fourth-generation techniques. Clinical outcomes were assessed through pain and functional scores, while radiographic parameters were analysed with a minimum follow-up of two years.

## Methods

### Study design

This retrospective case series included patients who underwent minimally invasive revision surgery for hallux valgus using either the third-generation (MICA) technique or the fourth-generation (META) technique, following failure of a previous correction (open or minimally invasive).

### Setting

Between January 2019 and August 2023, a series of 27 patients (33 feet) diagnosed with recurrent hallux valgus underwent revision surgical correction using third- or fourth-generation minimally invasive techniques (MICA or META).

All procedures were conducted at the same medical centre by a team of experienced surgeons specialising in minimally invasive surgery (M.V.P.F., G.F.F.) located in São Paulo, Brazil. Surgical intervention was considered only after conservative treatments such as footwear modification, physiotherapy, and specific guidelines had proven ineffective.

### Participants

The inclusion criteria were as follows: (1) patients aged 18 years or older; (2) the presence of mild, moderate, or severe recurrent hallux valgus, defined as a hallux valgus angle (HVA) greater than 20° and without associated metatarsus adductus; and (3) those who underwent surgical correction with either the MICA or META technique and completed a minimum follow-up of two years.

The exclusion criteria were as follows: patients with pre-existing degenerative foot or ankle conditions; patients with prior foot or ankle surgeries other than previous hallux valgus correction; and patients with residual sequelae from tibial pilon, ankle, or foot fractures. Additionally, patients with first tarsometatarsal joint instability confirmed clinically or radiographically with concurrent symptomatic flatfoot deformity requiring hindfoot reconstruction, were not included, as these conditions would represent indications for a different surgical strategy beyond the scope of this series.

### Ethical approval

The study was approved by the local ethics committee and conducted in accordance with the principles of the Declaration of Helsinki and the Guidelines for Good Clinical Practice. Reporting followed the STROBE recommendations for observational studies [14].

### Outcome measures

Clinical data included patient demographics (age, gender and body mass index), as well as follow-up duration of clinical evaluation. Functional outcomes were evaluated using the Manchester-Oxford Foot Questionnaire (MOXFQ) [15], the pain Visual Analogue Scale (VAS) to capture overall patient experience.

Radiographic analysis was performed on weightbearing anteroposterior foot radiographs. Preoperative and postoperative measurements included the hallux valgus angle (HVA), the intermetatarsal angle (IMA) and forefoot width (bone and soft tissue). Radiographic assessments were carried out at the last appointment.

Complications, including intraoperative fractures, hardware-related issues, loss of alignment, wound-healing problems, and other adverse events, were documented.

### Data collection

Study data were collected and managed using REDCap electronic data capture tools hosted at the Instituto Prevent Senior. REDCap (Research Electronic Data Capture) is a secure, web-based application designed to support data capture for research studies [16].

### Surgical procedure

The patients underwent surgery in the supine position without the use of a tourniquet, under spinal anaesthesia with sedation, in accordance with the institutional protocol. All procedures followed a day-hospital model, with admission and discharge occurring on the same day. The osteotomy was performed using either the third-generation MICA technique, based on a percutaneous chevron cut, or the fourth-generation META technique, employing a straight osteotomy [5, 12, 17]. Fixation was consistently achieved by engaging the lateral cortex of the proximal fragment, with rotational correction performed manually or with the assistance of a Kirschner wire when necessary [18].

An Akin osteotomy was undertaken in all cases using a minimally invasive approach, without screw fixation, and the medial eminence was excised according to the described technique [3, 19, 20].

**Fig. 1:**
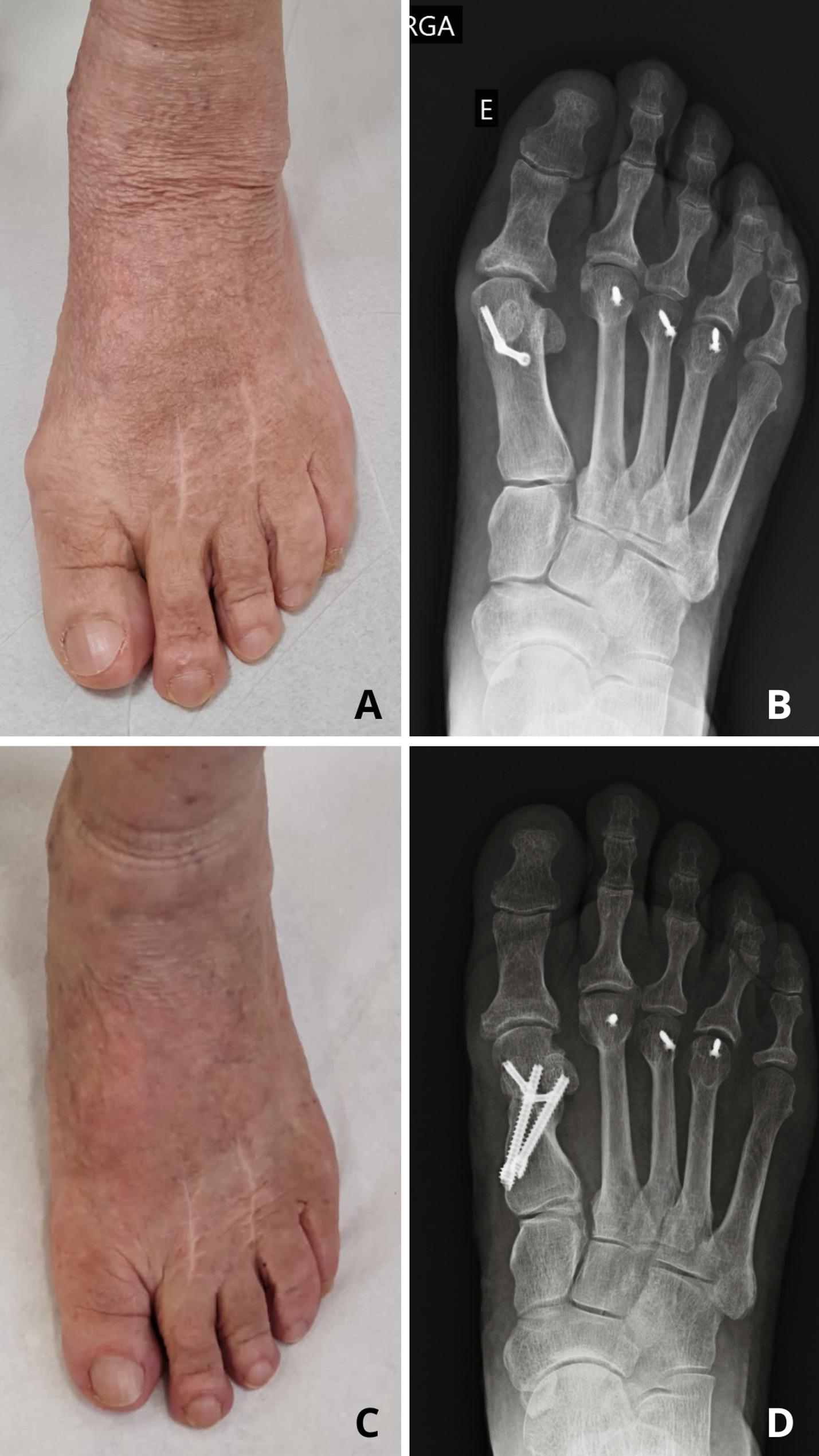
Clinical photographs and weightbearing anteroposterior (AP) radiographs. **A** Preoperative clinical image. **B** Preoperative AP radiograph. **C** Postoperative clinical image. **D** Postoperative AP radiograph after minimally invasive revision

In certain cases, implants were removed at the discretion of the operating surgeon. Furthermore, both the osteotomy and fixation were performed using two screws through a minimally invasive approach, thereby avoiding interference with previous fixation (Fig. 1).

### Closure

At the end of the procedure, the surgical incisions were irrigated with saline solution, and the skin was closed with 4 − 0 nylon sutures. A sterile gauze dressing was then applied, followed by an elastic bandage.

### Postoperative care

All patients were permitted immediate weightbearing using a rigid-soled sandal for a period of six weeks. Dressing changes were carried out weekly by the medical team during the first four weeks. Following the final dressing change, patients were instructed to commence toe mobilisation exercises, with particular emphasis on the hallux, to minimise postoperative stiffness.

### Statistical analysis

Continuous variables were expressed as means and standard deviations or medians and interquartile ranges, according to distribution. Normality was assessed with the Shapiro–Wilk test. Paired pre- and postoperative comparisons were performed using paired t-tests for normally distributed data and Wilcoxon signed-rank tests for non-parametric data.

Subgroup analyses comparing MICA and META were conducted separately. For continuous delta values (postoperative minus preoperative change), Welch’s t-test was applied when the assumption of normality was satisfied, while the Mann–Whitney test was used otherwise. A p-value of less than 0.05 was considered statistically significant. All analyses were performed in R [21].

## Results

A total of 33 consecutive cases (operated feet) from 27 patients were analysed, comprising 22 META cases (66.7%) and 11 MICA cases (33.3%). Six patients underwent bilateral procedures. The mean age was 66.7 ± 7.5 years (range, 50–86), with 97% of patients being female. The mean body mass index was 26.3 ± 5.1 kg/m², and the mean follow-up was 3.2 ± 1.3 years.

### Clinical outcomes

There were statistically significant improvements across all clinical measures following revision surgery. The mean VAS decreased from 8.3 ± 2.0 to 2.2 ± 2.9 (*p* < 0.001). All MOXFQ domains showed marked reduction: Walking/Standing decreased from 64.3 ± 23.2 to 21.1 ± 27.6, Pain from 67.4 ± 17.9 to 23.0 ± 27.2, and Social Interaction from 59.5 ± 26.7 to 24.8 ± 26.8. The MOXFQ Index improved from 64.1 ± 19.7 to 22.6 ± 26.0 (all *p* < 0.001). These changes are detailed in Table 1.

**Table 1:** Clinical and radiographic outcomes

Outcome	Preoperative			Postoperative			p value
	Mean ± SD	Median (IQR)	95% CI	Mean ± SD	Median (IQR)	95% CI	
HVA	31.8 ± 6.2	33.0 (7.0)	29.6 to 34.1	10.1 ± 7.8	9.5 (10.5)	7.3 to 13.0	< 0.001
IMA	13.1 ± 3.1	12.0 (5.0)	12.0 to 14.2	7.0 ± 3.6	6.0 (4.0)	5.7 to 8.3	< 0.001
Bone Forefoot Width	9.04 ± 0.86	9.09 (0.80)	8.73 to 9.35	8.48 ± 0.76	8.52 (0.92)	8.19 to 8.77	0.001
Soft-Tissue Forefoot Width	10.26 ± 0.87	10.33 (0.97)	9.95 to 10.58	9.87 ± 0.78	9.95 (0.92)	9.57 to 10.18	0.002
VAS	8.3 ± 2.0	9.0 (2.0)	7.6 to 9.0	2.2 ± 2.9	0.0 (4.0)	1.2 to 3.3	< 0.001
MOXFQ — Pain	67.4 ± 17.9	65.0 (20.0)	61.1 to 73.8	23.0 ± 27.2	15.0 (45.0)	13.4 to 32.7	< 0.001
MOXFQ — Walking/Standing	64.3 ± 23.2	67.9 (28.6)	56.0 to 72.5	21.1 ± 27.6	7.1 (35.7)	11.3 to 30.9	< 0.001
MOXFQ — Social	59.5 ± 26.7	62.5 (25.0)	50.0 to 68.9	24.8 ± 26.8	18.8 (37.5)	15.3 to 34.3	< 0.001
MOXFQ — Index	64.1 ± 19.7	62.5 (28.1)	57.1 to 71.0	22.6 ± 26.0	17.2 (32.8)	13.4 to 31.9	< 0.001

*Abbreviations*: *HVA* Hallux valgus angle, *IMA* Intermetatarsal angle, *VAS* Visual analogic pain scale, *MOXFQ* Manchester Oxford Foot Questionnaire, *SD* Standard deviation, *IQR* Interquartile range, *CI* Confidence interval

### Radiographic outcomes

Radiographic parameters demonstrated consistent and statistically significant correction. The mean HVA decreased from 31.8 ± 6.2° to 10.1 ± 7.8° (mean correction 21.8°, *p* < 0.001), while the IMA decreased from 13.1 ± 3.1° to 7.0 ± 3.6° (mean correction 6.1°, *p* < 0.001). Forefoot bone width was reduced from 9.0 ± 0.9 cm to 8.5 ± 0.8 cm (*p* = 0.001), and soft-tissue width decreased from 10.3 ± 0.9 cm to 9.9 ± 0.8 cm (*p* = 0.002).

**Fig. 2:**
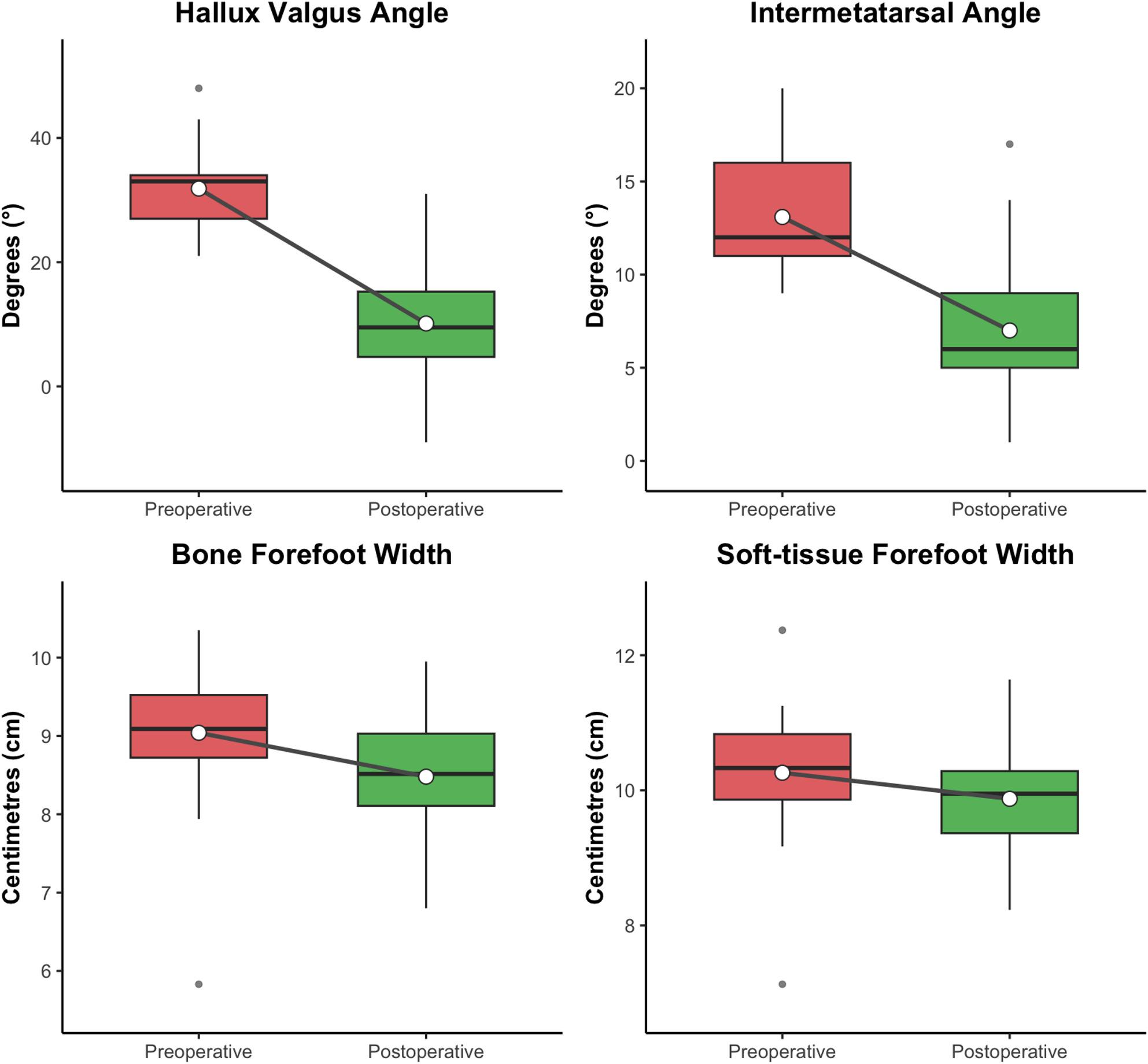
Preoperative and postoperative boxplots of HVA, IMA, bone forefoot width, and soft-tissue forefoot width on weightbearing anteroposterior radiographs; all improved (p < 0.01)

The graphical distribution of radiographic and clinical improvements is illustrated in Fig. 2.

### Subgroup analysis: MICA versus META

**Fig. 3:**
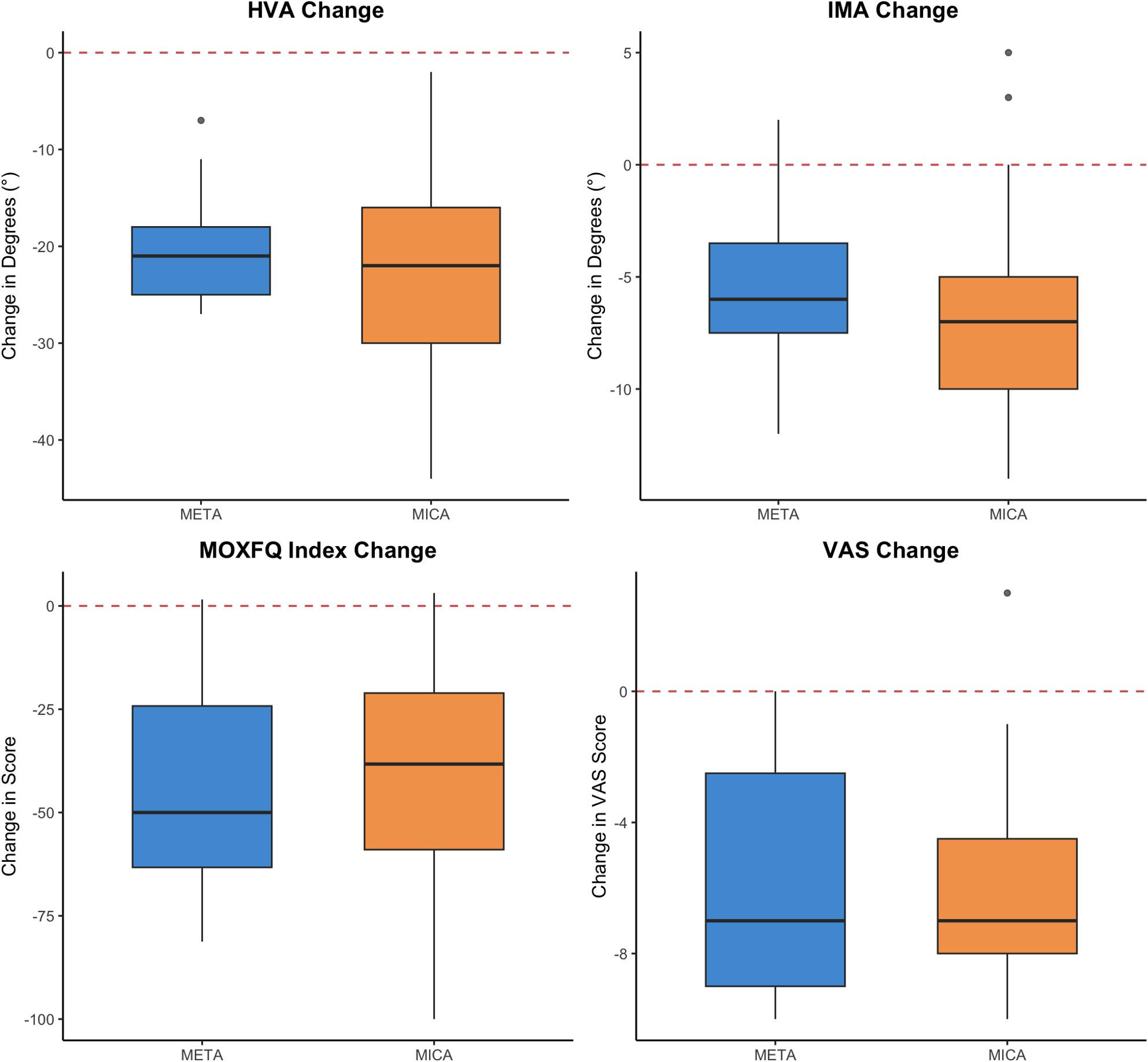
Subgroup analysis of changes (Δ) in HVA, IMA, MOXFQ Index, and VAS comparing MICA with META; no between-group differences (all p > 0.3)

No statistically significant differences were identified in changes between groups for clinical or radiographic outcomes. The mean correction of HVA was − 22.8 ± 9.9° in the MICA group and − 20.1 ± 6.4° in the META group (*p* = 0.364). The mean correction of IMA was − 6.3 ± 5.0° in the MICA group and − 5.8 ± 4.0° in the META group (*p* = 0.755). Likewise, changes in VAS, MOXFQ domains, and forefoot widths did not differ significantly (all *p* > 0.3) – Fig. 3.

These comparative findings are illustrated in Table 2.

**Table 2:** Comparison of clinical and radiographic parameters between MICA and META techniques

Outcome	MICA		META		*p *value
	Δ Mean ± SD	Δ Median (IQR)	Δ Mean ± SD	Δ Median (IQR)	
HVA	-22.8 ± 9.9	-22.0 (14.0)	-20.1 ± 6.4	-21.0 (7.0)	0.364
IMA	-6.3 ± 5.0	-7.0 (5.0)	-5.8 ± 4.0	-6.0 (4.0)	0.755
Bone Forefoot Width	-0.52 ± 0.74	-0.58 (0.81)	-0.61 ± 0.55	-0.61 (0.42)	0.719
Soft-Tissue Forefoot Width	-0.36 ± 0.67	-0.36 (0.56)	-0.42 ± 0.26	-0.34 (0.30)	0.731
VAS	-6.2 ± 3.6	-7.0 (3.5)	-5.7 ± 3.6	-7.0 (6.5)	0.832
MOXFQ — Pain	-42.0 ± 30.5	-42.5 (47.5)	-49.1 ± 22.3	-50.0 (32.5)	0.458
MOXFQ — Walking/Standing	-42.0 ± 30.3	-37.5 (42.9)	-45.5 ± 31.0	-57.1 (48.2)	0.767
MOXFQ — Social	-35.5 ± 28.2	-31.2 (31.2)	-33.0 ± 33.1	-50.0 (56.2)	0.829
MOXFQ — Index	-40.4 ± 28.4	-38.3 (37.9)	-43.5 ± 25.9	-50.0 (39.1)	0.760

*Abbreviations*: *HVA* Hallux valgus angle, *IMA* Intermetatarsal angle, *VAS* Visual analogic pain scale, *MOXFQ* Manchester Oxford Foot Questionnaire, *SD* Standard deviation, *IQR* Interquartile range, *CI* Confidence interval

### Associated procedures

Distal Metatarsal Metaphyseal Osteotomy (DMMO) was performed in 18.2% of cases. The procedure was undertaken on the central rays (second, third, and fourth metatarsals) to address metatarsalgia and to restore the metatarsal parabola. In patients who had previously undergone an open Weil osteotomy, the DMMO was carried out without removal of the existing screws.

### Patient satisfaction and complications

Overall, 28/33 cases (84.8%) were judged satisfactory with the revision procedure and indicated that they would undergo the same operation again. A first-metatarsal fracture with consequent loss of alignment occurred in one patient, who declined additional surgery.

Two additional patients required hardware removal, and in one case a screw breakage was observed; however, this did not result in loss of alignment and the patient remained asymptomatic.

### Primary surgical techniques

In the primary operations, a heterogeneous range of techniques had been utilised. Distal Chevron osteotomy combined with Akin osteotomy was the most frequently performed open procedure, undertaken in nine cases. Open isolated exostectomy was also carried out in six cases, whilst a further six feet underwent minimally invasive exostectomy combined with Akin osteotomy. Minimally invasive Reverdin osteotomy with Akin was performed in six cases. Two patients had previously undergone open Lapidus arthrodesis, and two further cases were treated with open basal osteotomy associated with Akin. Less frequently, one case had been managed with a minimally invasive Reverdin osteotomy combined with a basal osteotomy, and one case with an open Scarf osteotomy combined with Akin. This distribution reflects the considerable diversity of surgical strategies historically employed for the primary correction of hallux valgus within this cohort.

## Discussion

Our case series demonstrated significant clinical and radiographic improvement following revision of recurrent hallux valgus using third-generation (MICA) or fourth-generation (META) minimally invasive techniques, with consistent angular correction of HVA and IMA, together with improvements in MOXFQ scores and pain. In addition, our results demonstrated a high rate of patient satisfaction and a low incidence of complications, comparable to or exceeding that reported in other revision series employing open techniques [2, 13].

In recent years, third- and fourth-generation minimally invasive techniques have shown a marked increase in the number of publications, paralleling the overall growth of the scientific literature on hallux valgus [22]. This trend can be attributed to the high success rate, the low incidence of complications, and the reproducibility of the technique among foot and ankle surgeons worldwide [23].

Despite the extensive and robust literature regarding primary cases, there is a paucity of evidence with respect to the use of MICA or META techniques for the correction of recurrent hallux valgus.

Lewis et al. reported a case series describing a fourth-generation surgical technique for the revision of recurrent hallux valgus deformity, presenting preliminary outcomes [13]. In a series of 34 cases, the authors reported satisfactory clinical and radiographic outcomes (*p* < 0.05).

They demonstrated a reduction in the MOXFQ Index of 34.8 points, whereas our study achieved a reduction of 41.5 points, both with statistical significance. This improvement was also observed in angular correction, as evidenced by the analysis of HVA and IMA.

However, in our cohort we identified one case of metatarsal fracture with substantial loss of alignment and correction of the deformity. This finding may be explained by the older age profile of our patients and the associated bone fragility, which increases the risk of metatarsal fracture.

Indeed, our group recently demonstrated that each additional year of age independently increases the odds of first metatarsal fracture by 8.6% following minimally invasive hallux valgus surgery (OR 1.086; 95% CI 1.032–1.150; *p* = 0.003) [24, 25].

About the risk of metatarsal fracture, in theory the META technique may reduce the likelihood of lateral cortical fracture, as it provides a greater amount of bone available for fixation when compared with the Chevron osteotomy (MICA) [5, 25].

However, our recently published case-control study found no statistically significant difference in fracture risk between the two techniques (7.2% for MICA vs. 4.1% for META; *p* = 0.167), suggesting that patient-related factors, particularly advanced age, may carry greater weight than the choice of osteotomy geometry alone [25].

It is important to emphasise that both the MICA and META techniques are based on the same biomechanical principle: translation of the metatarsal head, correction of pronation, and fixation with screws, with at least one screw engaging the lateral cortex to ensure optimal conditions for bone healing [11, 12, 17, 26].

Whilst both techniques demonstrate broad applicability in the revision setting, certain clinical and anatomical factors should be considered relative contraindications. No absolute contraindications specific to revision minimally invasive hallux valgus surgery have been formally established; however, Vernois and Redfern proposed that a first-to-second intermetatarsal space-to-metatarsal head width ratio exceeding 1 may preclude adequate lateral translation with a distal osteotomy, favouring a proximal basal approach in such cases [17].

Our findings align with those of Magnan et al. [27] who reported favourable outcomes using percutaneous distal osteotomy for recurrent hallux valgus. Their study demonstrated significant radiographic correction, with HVA improving from 26.1° to 9.7° and IMA from 11.5° to 6.7°. Whilst our investigation achieved greater mean HVA correction (21.8° versus 16.4°), both studies confirm the efficacy of minimally invasive techniques in revision surgery.

Magnan et al. reported a longer follow-up of 9.8 years compared to our 3.2 years, providing robust evidence for correction durability. Although different outcome measures preclude direct comparison, both investigations demonstrated substantial improvements in pain and function.

Both studies reported similarly low complication rates, supporting the safety of minimally invasive revision surgery. Magnan et al. [27] documented a 3.1% re-recurrence rate with minor complications including delayed union and reduced joint motion. Our series observed comparable adverse events, including one metatarsal fracture and two hardware removals, with 84.8% patient satisfaction.

The older demographic in our cohort (66.7 versus 54.2 years) did not adversely influence outcomes. These results collectively strengthen evidence supporting third and fourth-generation MIS techniques as reliable treatment for recurrent hallux valgus.

When contrasted with primary-case data from Lam et al. (729 feet treated with a fourth-generation percutaneous transverse osteotomy), our revision series achieved a comparable magnitude of angular correction and clinically meaningful functional gains. Mean HVA correction was 21.8° in our cohort versus 22.2° in Lam et al., and IMA correction was 6.1° versus 8.4°, respectively. MOXFQ Index improved from 64.1 to 22.6 in our series (Δ 41.5) compared with 36.9 to 13.4 in the primary cohort (Δ 23.5) [12].

Although cross-study comparisons should be interpreted with caution given differing baselines and eligibility criteria, these findings indicate that third- and fourth-generation MIS techniques remain effective in the revision setting, delivering angular correction and functional improvement that approach those observed in primary surgery.

### Generalisability

The findings of this study may be generalised to tertiary centres with established expertise in percutaneous surgery, although they may not be directly extrapolated to units without a consolidated learning curve. The patient cohort was composed predominantly of women of advanced age with a mean body mass index within the overweight range, which reflects the demographic profile typically encountered in clinical practice. The outcomes observed suggest that minimally invasive surgery represents a reproducible and effective option for revision procedures in appropriately selected cases.

### Strengths

This study presents several strengths. It constitutes the first reported series of recurrent hallux valgus revisions performed using third- and fourth-generation minimally invasive techniques. Clinical outcomes were systematically assessed with validated instruments, including the MOXFQ and VAS, ensuring methodological robustness. All patients were treated in a real-world clinical setting, thereby enhancing the external relevance of the findings. Furthermore, procedures were performed at a single centre by the same surgical team, with standardised operative methods and postoperative protocols, which minimises variability and strengthens the internal validity of the study.

### Limitations

This study has several limitations that must be acknowledged. It is a retrospective case series without a control group, precluding direct comparison with outcomes of primary hallux valgus correction. The sample size was relatively small (33 feet), which limits the statistical power of subgroup analyses. The mean follow-up of three years may be insufficient to capture late recurrences, which are known to occur after five to ten years. Radiographic measurements were obtained from two-dimensional radiographs, without three-dimensional assessment such as weightbearing CT, which could provide more detailed information on deformity correction. No formal evaluation of general health-related quality of life was undertaken beyond the disease-specific questionnaires. Finally, the cohort included patients with a wide range of different index procedures for hallux valgus correction, introducing potential heterogeneity.

### Interpretation

This study demonstrated significant clinical and radiographic improvements following revision of recurrent hallux valgus using minimally invasive techniques (MICA and META). Consistent angular correction of both HVA and IMA was achieved, accompanied by marked improvement in MOXFQ scores and pain levels. Patient satisfaction was high, and the complication rate was low, findings that are comparable to, or exceed, those reported in revision series employing open techniques. The results also highlight the importance of adhering to the principles of fixation with two screws in both MICA and META, and of preserving existing implants whenever feasible, as a safe and effective surgical strategy.

### Clinical implications and future research

This study provides evidence that third- and fourth-generation MIS techniques can be safely applied in the revision setting as well as in primary procedures. Prospective, multicentre studies with larger cohorts and longer follow-up are warranted to confirm the durability of these results and to better define recurrence rates over time. Future research should also address the influence of anatomical factors, such as first-ray length and metatarsus adductus, as well as the potential role of emerging technologies, including three-dimensional surgical guides and predictive artificial intelligence, in reducing recurrence and complications.

## Conclusion

Third- and fourth-generation minimally invasive surgical techniques have proven effective in correcting radiographic parameters and improving pain and functional scores in patients undergoing revision for recurrent hallux valgus, with a low rate of complications.

## Supplementary Information

Supplementary Material 1.

Supplementary Material 2.

Supplementary Material 3.

Supplementary Material 4.

Supplementary Material 5.

Supplementary Material 6.

Supplementary Material 7.

Supplementary Material 8.

Supplementary Material 9.

Supplementary Material 10.

Supplementary Material 11.

## Data Availability

The datasets generated and/or analysed during the current study are not publicly available due to ethical and privacy restrictions but are available from the corresponding author on reasonable request.

## References

[1] de Carvalho KAM, Baptista AD, de Cesar Netto C, Johnson AH, Dalmau-Pastor M. Minimally Invasive Chevron-Akin for Correction of Moderate and Severe Hallux Valgus Deformities: Clinical and Radiologic Outcomes With a Minimum 2-Year Follow-up. Foot Ankle Int. 2022;43(10):1317–30. 36000192 10.1177/10711007221114123

[2] Ferreira GF, Borges VQ, Moraes LVM, Stefani KC. Percutaneous Chevron/Akin (PECA) versus open scarf/Akin (SA) osteotomy treatment for hallux valgus: A systematic review and meta-analysis. PLoS ONE. 2021;16(2):e0242496. 33596196 10.1371/journal.pone.0242496 PMC7888602

[3] Ferreira GF, Nunes GA, Pugliese GM, Dinato MCME, Lewis TL, Sato G, et al. Minimally invasive Chevron-Akin (MICA) osteotomies without Akin fixation in hallux valgus correction: a case series with 2-year follow-up. Eur J Orthop Surg Traumatol. 2024;34(5):2339–45. 10.1007/s00590-024-03924-8. Epub 2024 Apr 7. PMID: 38583122. 10.1007/s00590-024-03924-8 38583122

[4] Lee M, Walsh J, Smith MM, Ling J, Wines A, Lam P. Hallux Valgus Correction Comparing Percutaneous Chevron/Akin (PECA) and Open Scarf/Akin Osteotomies. Foot Ankle Int. 2017;38(8):838–46. 28476096 10.1177/1071100717704941

[5] Lewis TL, Lau B, Alkhalfan Y, Trowbridge S, Gordon D, Vernois J, Lam P, Ray R. Fourth-Generation Minimally Invasive Hallux Valgus Surgery With Metaphyseal Extra-Articular Transverse and Akin Osteotomy (META): 12 Month Clinical and Radiologic Results. Foot Ankle Int. 2023;44(3):178–91. 36788732 10.1177/10711007231152491

[6] Lewis TL, Ray R, Miller G, Gordon DJ. Third-Generation Minimally Invasive Chevron and Akin Osteotomies (MICA) in Hallux Valgus Surgery: Two-Year Follow-up of 292 Cases. J Bone Joint Surg Am. 2021;103(13):1203–11. 33764936 10.2106/JBJS.20.01178 PMC8265548

[7] Lewis TL, Ray R, Robinson P, Dearden PMC, Goff TJ, Watt C, Lam P. Percutaneous Chevron and Akin (PECA) Osteotomies for Severe Hallux Valgus Deformity With Mean 3-Year Follow-up. Foot Ankle Int. 2021;42(10):1231–40. 34111991 10.1177/10711007211008498

[8] Lewis TL, Robinson PW, Ray R, Dearden PMC, Goff TAJ, Watt C, Lam P. Five-Year Follow-up of Third-Generation Percutaneous Chevron and Akin Osteotomies (PECA) for Hallux Valgus. Foot Ankle Int. 2023;44(2):104–17. 36692121 10.1177/10711007221146195

[9] Lam P, Fletcher L, Watt C, Ray R, Dalmau-Pastor M, de Cesar Netto C, Lewis TL. First Metatarsal Pronation Correction After Fourth-Generation Percutaneous Transverse Osteotomy for Hallux Valgus. Foot Ankle Int. 2025:10711007251344273. 10.1177/10711007251344273 40580076

[10] Ferreira GF, Mattos EDMC, Santos TFD, Miziara P, Pereira MV. Can the Percutaneous Chevron and Akin (Peca) Technique Correct the Pronation of the First Metatarsal in Hallux Valgus? Acta Ortop Bras. 2023;31(spe2):e265206. 10.1590/1413-785220233102e265206. PMID: 37323155; PMCID: PMC10263439. 37323155 10.1590/1413-785220233102e265206 PMC10263439

[11] Lewis TL, Barakat A, Mangwani J, Ramasamy A, Ray R. Current concepts of fourth-generation minimally invasive and open hallux valgus surgery. Bone Joint J. 2025;107–B(1):10–8. 39740690 10.1302/0301-620X.107B1.BJJ-2024-0597.R2

[12] Lam P, Murphy EP, Chua MJ, Ray R, Watt C, Robinson PW, et al. Fourth-Generation Percutaneous Transverse Osteotomies for Hallux Valgus. J Bone Joint Surg Am. 2025. 10.2106/JBJS.24.01326 PMC12462676 40854004

[13] Lewis TL, Ray R, Lam P. Revision of Recurrent Hallux Valgus Deformity Using a Percutaneous Distal Transverse Osteotomy: Surgical Considerations and Early Results. Foot Ankle Clin. 2025;30(2):375–84. 40348469 10.1016/j.fcl.2024.04.010

[14] von Elm E, Altman DG, Egger M, Pocock SJ, Gotzsche PC, Vandenbroucke JP, Initiative S. The Strengthening the Reporting of Observational Studies in Epidemiology (STROBE) statement: guidelines for reporting observational studies. PLoS Med. 2007;4(10):e296. 17941714 10.1371/journal.pmed.0040296 PMC2020495

[15] Dawson J, Doll H, Coffey J, Jenkinson C, Oxford, Birmingham F, Ankle Clinical Research G. Responsiveness and minimally important change for the Manchester-Oxford foot questionnaire (MOXFQ) compared with AOFAS and SF-36 assessments following surgery for hallux valgus. Osteoarthritis Cartilage. 2007;15(8):918–31. 17383907 10.1016/j.joca.2007.02.003

[16] Harris PA, Taylor R, Thielke R, Payne J, Gonzalez N, Conde JG. Research electronic data capture (REDCap)--a metadata-driven methodology and workflow process for providing translational research informatics support. J Biomed Inf. 2009;42(2):377–81. 10.1016/j.jbi.2008.08.010 PMC2700030 18929686

[17] Vernois J, Redfern DJ. Percutaneous Surgery for Severe Hallux Valgus. Foot Ankle Clin. 2016;21(3):479–93. 27524702 10.1016/j.fcl.2016.04.002

[18] Ferreira GF, Nunes GA, Dorado DS, Dinato M, Lewis TL, Ray R, Filho MVP. Correction of First Metatarsal Pronation in Metaphyseal Extra-articular Transverse Osteotomy for Hallux Valgus Correction. Foot Ankle Orthop. 2023;8(3):24730114231198527. 37736327 10.1177/24730114231198527 PMC10510346

[19] Ferreira GF, Nunes GA, Mattos EDMC, Pedroso JP, Lewis TL, Lam P, Filho MVP. Technique Tip: Medial prominence bone spur resection in the third-generation percutaneous Chevron-Akin Osteotomy Technique (PECA) for hallux valgus correction. Foot Ankle Surg. 2022;28(4):460–3. 34838427 10.1016/j.fas.2021.11.004

[20] Ferraz Ferreira G, Araujo Nunes G, Fiorin da Costa G, Mattos EDMC, Lorchan Lewis T, Ray R, Alves Patriarcha V, Carvalho P, Viana Pereira Filho M. Is Akin fixation necessary in the Percutaneous Chevron and Akin osteotomies (PECA) technique? A retrospective comparative study with 2-year follow-up. Eur J Orthop Surg Traumatol. 2025;35(1):212. 40407922 10.1007/s00590-025-04316-2

[21] Team RC. R: A language and environment for statistical computing. In. Vienna, Austria: R Found. 2020.

[22] Ferreira GF, Stefani KC. A Global Bibliometric Analysis of Hallux Valgus Research (1999–2019). J Foot Ankle Surg. 2021;60(3):501–6. 33573904 10.1053/j.jfas.2020.09.016

[23] Toepfer A, Strassle M. The percutaneous learning curve of 3rd generation minimally-invasive Chevron and Akin osteotomy (MICA). Foot Ankle Surg. 2022;28(8):1389–98. 35882575 10.1016/j.fas.2022.07.006

[24] Pereira Filho MV, Stefani KC, Ferreira GF, Nogueira MP. Risk Factors Associated With Foot and Ankle Insufficiency Fractures in Postmenopausal Sedentary Women. Foot Ankle Int. 2021;42(4):482–7. 33203230 10.1177/1071100720969654

[25] Ferreira GF, Proenca DS, Araujo GF, Porto RS, Sevilla D, Araujo Nunes G, Mattos EDMC, Lewis TL, Ray R, Pereira Filho MV. Risk factors for first metatarsal fracture in minimally invasive hallux valgus surgery: a case-control study. Bone Joint J. 2026;108–B(3):361–8. 41763261 10.1302/0301-620X.108B3.BJJ-2025-0253.R1

[26] Lewis TL, Mansur H, Ferreira GF, Filho MVP, Battaglion LR, Zambelli R, Ray R, Nunes GA. Comparative biomechanical study of different screw fixation methods for minimally invasive hallux valgus surgery: A finite element analysis. Foot Ankle Surg. 2025;31(2):160–9. 39261184 10.1016/j.fas.2024.09.001

[27] Magnan B, Negri S, Maluta T, Dall’Oca C, Samaila E. Minimally invasive distal first metatarsal osteotomy can be an option for recurrent hallux valgus. Foot Ankle Surg. 2019;25(3):332–9. 29409172 10.1016/j.fas.2017.12.010

